# N6-methyladenosine methylation in acute lung injury: Mechanisms and research progress

**DOI:** 10.1016/j.jointm.2025.07.001

**Published:** 2025-08-20

**Authors:** Yating Hu, Yijie Wang, Xiyue Liu, Xiaoli Xue, Binbin Li, Fangwei Li

**Affiliations:** Department of Respiratory and Critical Care Medicine, Lanzhou University Second Hospital, Lanzhou University, Lanzhou, Gansu, China

**Keywords:** Acute lung injury, m6A RNA Methylation, RNA Methylations, Sepsis, Acute respiratory distress syndrome

## Abstract

N6-methyladenosine (m6A) methylation is the most prevalent and abundant internal post-transcriptional RNA modification in eukaryotic cells, playing an important regulatory role in various biological processes. The biological functions of m6A modification are dynamically and reversibly mediated by methyltransferases (writers), demethylases (erasers), and m6A binding proteins (readers). Acute lung injury (ALI) is a common critical condition characterized by diffuse edema within the pulmonary interstitium and alveoli, and is associated with high morbidity and mortality. Recent studies have identified that aberrant expression of m6A regulators is closely associated with ALI development. This review highlights the progress in research on m6A writers, erasers, and readers in ALI, focusing on their molecular regulatory mechanisms. Elucidating the molecular mechanisms of m6A and its associated proteins in ALI may reveal new therapeutic strategies and targets.

## Introduction

Acute lung injury (ALI) is a critical clinical condition characterized by diffuse inflammation of the pulmonary interstitium and intractable hypoxemia, typically caused by factors such as trauma, pneumonia, shock, and sepsis. Clinical symptoms of ALI include pulmonary edema, impaired gas exchange, and hypoxemia.[1], [2], [3], [4], [5]. The mortality rate among hospitalized patients with ALI is reportedly as high as 30%–45%.^[^^6^^,^^7^^]^ Currently, the treatment of ALI is centered on respiratory support therapy. In patients with severe ALI, mechanical ventilation provides effective respiratory support and improves oxygenation. Pharmacological management of ALI aims to alleviate clinical symptoms by suppressing excessive inflammation and reducing oxidative stress. Currently, corticosteroids are among the most widely used agents. They exert potent anti-inflammatory effects by inhibiting the release of inflammatory cytokines and reducing vascular permeability. Other agents, such as phosphodiesterase (PDE) inhibitors and neutrophil elastase inhibitors, have also demonstrated therapeutic potential due to their anti-inflammatory and antioxidant properties.^[^^2^^,^^8^^,^^9^^]^ Although the molecular mechanisms associated with ALI have been partially elucidated, its treatment remains extremely challenging, as ALI often arises in the complex clinical context of multi-organ failure. In addition, patients undergoing prolonged mechanical ventilation are at increased risk of developing pulmonary fibrosis, which further exacerbates disease severity and contributes to high mortality rates.^[^^10^^,^^11^^]^ Consequently, it is imperative to further elucidate the pathological mechanisms of ALI, especially its dynamic evolution within complex clinical scenarios, to inform the development of more effective future interventions.

Epigenetics focuses on heritable changes in gene expression regulation, and its scope encompasses DNA methylation, histone modifications, regulatory roles of non-coding RNAs, and the dynamic changes in chromatin structure. These mechanisms play multifaceted and important roles in cellular physiological functions and pathological states. RNA modification is an important component of mammalian epigenetic regulation, including chemical modifications such as methylation, phosphorylation, and acetylation. N6-methyladenosine (m6A) methylation is currently believed to be the most abundant RNA methylation modification in eukaryotes.^[^^12^^]^ In recent years, many studies have shown that m6A methylation regulates the function of immune cells in the inflammatory response and plays a crucial role in the pathogenesis of several respiratory diseases.^[^[13], [14], [15], [16], [17]^]^ This review examines the roles and molecular mechanisms of m6A methylation modifications in ALI and highlights the potential value of targeting m6A in ALI therapeutic strategies.

## Regulation of m6A Modification

m6A modification refers to the methylation of the nitrogen at the sixth position (N6) of the adenine base in RNA and predominantly occurs in long exon regions, stop codon regions, and the 3′-untranslated region (3′-UTR).^[^^18^^,^^19^^]^ m6A modifications regulate gene expression by affecting RNA translation, splicing, stabilization, and export. These processes are influenced by three important regulators: m6A methyltransferases (m6A writers), m6A demethylases (m6A erasers), and m6A-binding proteins (m6A readers).^[^^20^^]^ m6A writers catalyze the co transcriptional installation of m6A and primarily include methyltransferase-like 3 (METTL3), methyltransferase-like 14 (METTL14), Wilms tumor 1-associated protein (WTAP), virus-like methyltransferase-associated protein (KIAA1429/VIRMA), and CCCH-type zinc finger domain-containing protein 13 (ZC3H13). METTL3 was the first writer to be discovered and functions in a complex with METTL14, which primarily stabilizes the m6A methyltransferase complex.^[^^21^^,^^22^^]^ WTAP plays a crucial role in the selective splicing of RNA and the regulation of gene expression. As the only noncatalytic subunit in the m6A writer complex, WTAP anchors the WTAP-METTL3-METTL14 complex to nuclear speckles, thereby facilitating the dynamic regulation of m6A modifications.^[^^23^^]^ m6A-modified RNA is demethylated by m6A erasers, indicating that m6A modification is a dynamic and reversible process.^[^^24^^]^ The main known m6A erasers are fat mass obesity-associated protein (FTO) and ALKB homolog 5 (ALKBH5). As part of the reversible m6A methylation process, FTO regulates adipogenesis and energy homeostasis. ALKBH5-directed m6A demethylation is involved in splicing and the production of mRNAs with longer 3′-UTRs.^[^^25^^,^^26^^]^ m6A readers recognize and bind to m6A methylation sites, thereby regulating mRNA translation, splicing, stability, and nuclear export. The regulators involved include (1) the YTH domain protein family: YTHDF1-3 and YTHDC1Z-2; (2) heterogeneous nuclear ribonucleoproteins (hnRNPs): hnRNPC, hnRNPG, and hnRNPA2B; and (3) insulin-like growth factor 2 mRNA-binding proteins (IGF2BPs): IGF2BP1-3. YTHDF2 was the first identified m6A-binding protein and promotes mRNA degradation by binding to the 3′-UTR. Through its interaction with the translation initiation factor eIF3, YTHDF1 facilitates ribosome loading, thereby enhancing the translation efficiency of target proteins.^[^^27^^,^^28^^]^ YTHDC1 is a nuclear m6A reader, IGF2BP proteins are cytoplasmic m6A readers, and YTHDC2 exhibits a unique subcellular localization, being present in both the nucleus and the cytoplasm.^[^^20^^,^^29^^,^^30^^]^ hnRNPA2B1 acts as a nuclear m6A reader and plays an important role in telomere maintenance and DNA repair.^[^^31^^,^^32^^]^

## m6A Modification Regulators in ALI

**Figure 1:**
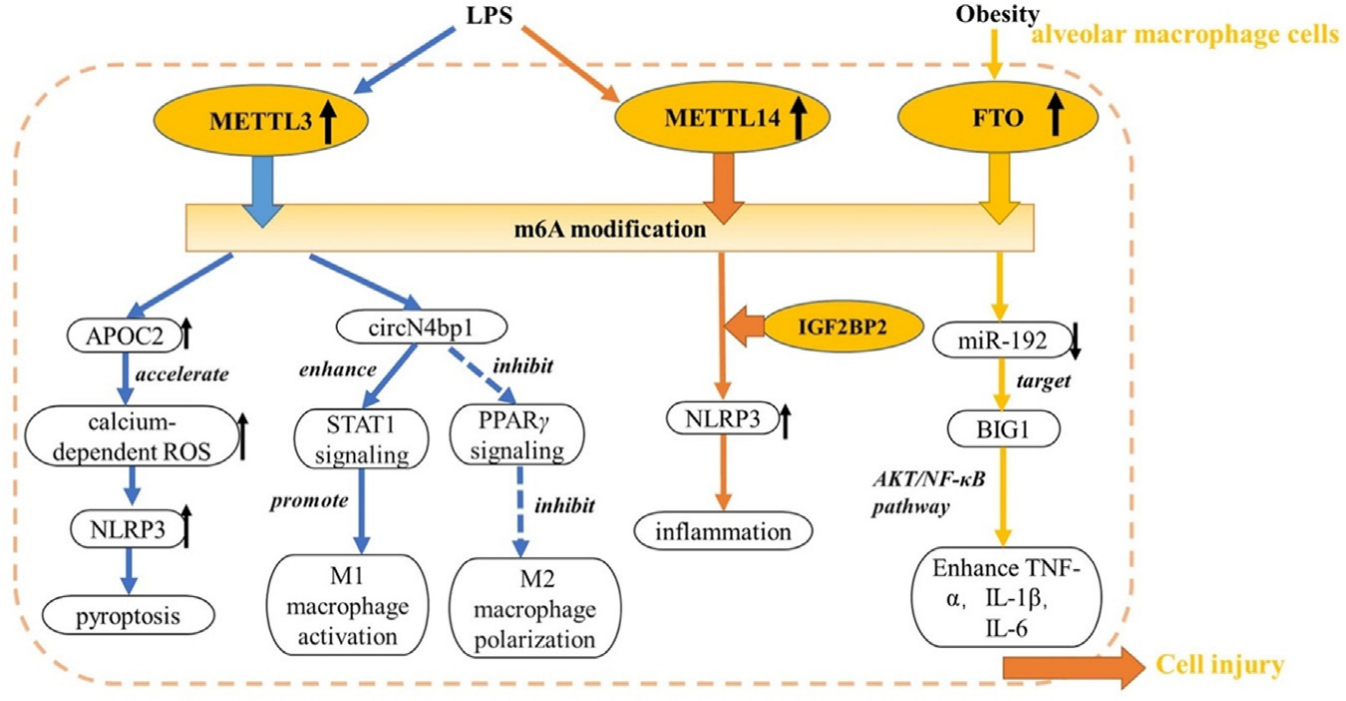
Role of m6A modification in alveolar macrophage cells. APOC2: Apolipoprotein 2; BIG1: Brefeldin a-inhibited guanine nucleotide exchange factor 1; FTO: Fat mass and obesity-associated protein; IGF2BP2: Insulin-like growth factor 2 mRNA binding protein 2; IL: Interleukin; LPS, Lipopolysaccharide; m6A: N6-methyladenosine; METTL: Methyltransferase-like; NLRP3: NACHT, LRR, and PYD domains-containing protein 3; PPAR: Peroxisome proliferator-activated receptor; ROS: Reactive oxygen species; STAT1: Signal transducer and activator of transcription 1; TNF: Tumor necrosis factor; ↑: Increase expression; ↓: Decrease expression.

**Figure 2:**
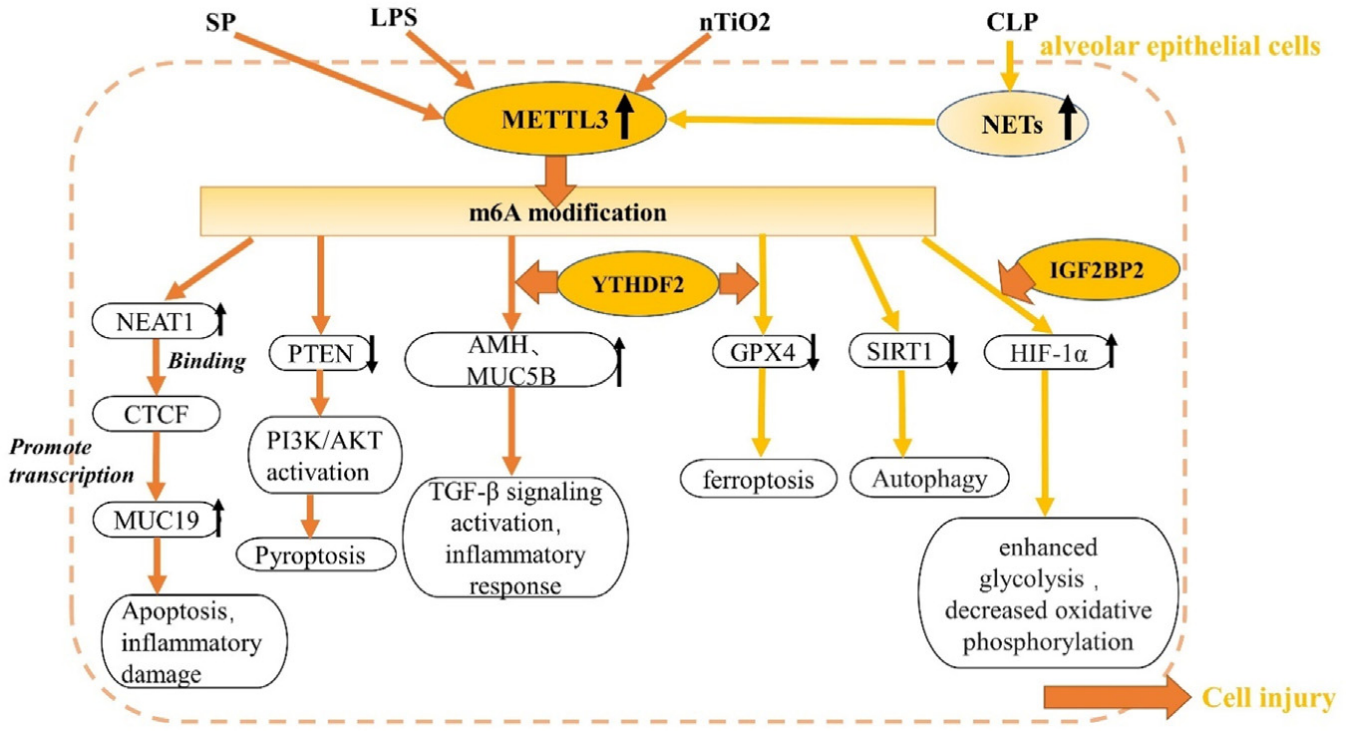
Role of m6A modification in alveolar epithelial cells. m6A, N6-methyladenosine. AKT: Protein kinase B (PKB); AMH: Anti-mullerian hormone; CTCF: CCCTC-binding factor; CLP: Cecal ligation and puncture; GPX4: Glutathione peroxidase-4; HIF-1α: Hypoxia-inducible factor-1α; IGFBP2: Insulin-like growth factor 2 mRNA binding protein 2; LPS: Lipopolysacchariden; METTL: Methyltransferase-like; MUC19: Mucin 19; MUC5B: Mucin 5B, oligomeric gel-forming; NEAT1: Nuclear-enriched abundant transcript 1; NETs: Neutrophil extracellular traps; PI3K: Phosphoinositide 3-kinase; PTEN: Phosphatase and tensin homolog deleted on chromosome ten; SIRT1: Silent information regulator sirtuin 1; SP: Streptococcus pneumoniae; TiO2: Nanoscale titanium dioxide; YTHDF: YT521-B homology domain family; ↑: Increase expression; ↓: Decrease expression.

Multiple studies have demonstrated that m6A methylation levels are significantly elevated in lipopolysaccharide (LPS)-induced ALI models compared with normal lung tissue, with enrichment primarily in the 3′-UTR and coding sequence, and accompanied by differential expression of key regulatory factors.^[^^33^^,^^34^^]^ These findings highlight the significance of m6A methylation in ALI progression and its potential as a therapeutic target (Figure 1, Figure 2).

### m6A writers in ALI

#### METTL3

The role of m6A modification in ALI development has garnered significant attention, with the writer protein METTL3 being the most extensively studied. Previous studies have demonstrated that METTL3 expression is significantly elevated in numerous lung injury models, both *in vivo* and *in vitro*. Mechanistically, METTL3 inhibits the differentiation of alveolar epithelial cell (AEC) type II (AECII). Moreover, METTL3 knockdown reduced apoptosis, inflammation, and cell death, while promoting the proliferation of AECs.^[^[35], [36], [37], [38]^]^ Numerous studies have confirmed that neutrophil extracellular traps (NETs), which are highly expressed in patients with ALI and sepsis-induced ALI mouse models, increase the level of m6A modification while inducing METTL3 upregulation, which exacerbates sepsis-induced lung injury by targeting glutathione peroxidase-4 (GPX4), hypoxia-inducible factor-1α, and Sirtuin 1 in AECs, thereby causing ferroptosis and impaired autophagic flux,^[^[39], [40], [41]^]^ suggesting METTL3-mediated m6A modification as a critical pathway through which NETs promote sepsis-induced ALI. Acute respiratory distress syndrome (ARDS) is a severe stage of ALI, characterized by histological features including pulmonary edema, hyaline membrane formation, alveolar hemorrhage, and inflammation, which eventually progresses to acute hypoxemic respiratory failure.^[^^1^^,^^42^^]^ Notably, METTL3 has also been implicated in the regulation of non-coding RNA (ncRNA) in sepsis-induced ARDS. For example, Zhao et al.^[^^43^^]^ showed that METTL3 upregulates circN4bp1 through m6A modification, promoting cecal ligation and puncture (CLP)-induced M1 macrophage activation in ALI mice, and driving ARDS progression. These findings highlight the dual regulatory role of m6A modifications in both the mRNA and ncRNA pathways during ALI.

However, Cui et al.^[^^44^^]^ reported that METTL3 expression is downregulated in LPS-induced lung tissues. METTL3 stabilizes miR-29a-3p via m6A modification, thereby inhibiting AEC apoptosis and inflammation. Other studies have shown that protein expression levels of METTL3 tend to decrease.^[^^33^^,^^45^^]^ Jin et al.^[^^46^^]^ further demonstrated that METTL3 knockdown in bronchial epithelial cells enhances pro-inflammatory cytokine secretion, contributing to ALI. In addition, Chen et al.^[^^47^^]^ showed that METT3 controls the tetratricopeptide repeat domain 4 gene by inhibiting mitochondrial damage via the heat shock protein 70/reactive oxygen species (ROS)/NLR family pyrin domain containing 3 (NLRP3) signaling pathway to reduce inflammation. In response to the inconsistency in METTL3 expression during the development of ALI, we posit that several factors may contribute to this phenomenon. First, m6A methylation modification exhibits significant dynamism. Therefore, studies collecting data at different post-modeling time-points (e.g., 6, 12, and 24 h) may reach conflicting conclusions. Second, METTL3 expression varies across lung cell types. It is upregulated in human pulmonary AECs (HPAEpiC), the mouse alveolar macrophage cell line, and RAW264.7 monocytes, but downregulated in the human bronchial epithelial cell line BEAS-2B and human umbilical vein and pulmonary microvascular endothelial cells. Furthermore, discrepancies exist in the methods used to construct ALI models across current studies, including LPS intraperitoneal injection, intratracheal instillation, and CLP surgery. The LPS concentration may critically affect cell responses. For example, Wang et al.^[^^37^^]^ reported that in AECII cells, LPS at 5–10 µg/mL reduces viability and proliferation in a dose-dependent manner, with maximal inhibition at 20 µg/mL. Finally, most studies did not report the number and frequency of cell passages. The phenotypic and molecular properties of primary cells may be altered during *in vitro* culture as the number of passages increases. Further investigation is required to determine whether METTL3 exerts protective or detrimental effects.

#### METTL4

Sang et al.^[^^48^^]^ found that METTL4 is differentially expressed in sepsis-induced ALI mice, based on m6A and RNA sequencing. METTL4 deletion reduced ferroptosis markers (lipid ROS, malondialdehyde, and Fe^2+^), altered GPX4 and Solute Carrier Family 7 Member 11 protein levels, and attenuated iron death in AECs. The mechanism involves METTL4-mediated nuclear factor erythroid 2-related factor 2 (Nrf2) 3′-UTR methylation, which allows YTHDF2 to recognize and bind to m6A methylated Nrf2, thereby promoting Nrf2 degradation. This leads to an increase in intracellular oxidative stress, which subsequently promotes ferroptosis.

#### METTL14

Blood METTL14 mRNA levels are significantly higher in patients with sepsis-induced ALI than in healthy volunteers.^[^^49^^]^ In LPS-stimulated A549 cells, METTL14 mRNA levels are significantly elevated. METTL14 knockdown reduced interleukin (IL)-18 and IL-1β levels, decreased LncRNA THRIL m6A modification in LPS-treated A549 cells, and inhibited autophagy levels, thereby suppressing LPS-induced ALI.^[^^50^^,^^51^^]^ Macrophage-mediated inflammation is crucial in ALI, and NLRP3 inflammasomes are a key factor in macrophage activation. Cao et al.^[^^52^^]^ found that METTL14 is pivotal for NLRP3 inflammasome activation. METTL14 knockdown in LPS-induced ALI mouse lung tissue significantly reduced NLRP3 protein expression. The alveolar structure in the ALI model lung tissues remained relatively intact, with less inflammatory cell infiltration and reduced pulmonary edema. Subsequently, m6A RNA immunoprecipitation confirmed that NLRP3 is a downstream target of METTL14.

### m6A erasers in ALI

#### FTO

Studies on FTO in ALI have shown that FTO knockdown, both *in vivo* and *in vitro*, reduces alveolar structural damage, tissue edema, lung inflammation, LPS-induced injury, and cell death in A549 cells.^[^^53^^]^ In a study on obesity and ALI/ARDS, Wu et al.^[^^54^^]^ observed that m6A methylation was reduced in the lung tissues and alveolar macrophages of obese mice, while FTO expression was increased. FTO overexpression, induced by obesity, reduced miR-192 production via m6A demethylation of pri-miR-192. Downregulation of miR-192 exacerbated the LPS-induced polarization of macrophages toward the M1 phenotype. Activation of the protein kinase B/nuclear factor kappa B inflammatory pathway aggravated lung tissue injury. These findings suggest that the role of m6A modifications in obesity-associated ALI/ARDS requires further investigation. In contrast, Zhao et al.^[^^55^^]^ demonstrated that FTO overexpression in LPS-stimulated RAW264.7 macrophages reduced inflammatory factors (tumor necrosis factor [TNF]-α, IL-1β, and IL-6) and prostaglandin E2 synthesis, attenuating ferroptosis. This discrepancy may be attributed to variations in LPS concentrations used across experimental models.

#### ALKBH5

A study on the regulatory mechanisms underlying ALI identified ALKBH5 as a regulator of circular RNA (circRNA) expression. In primary microvascular endothelial cells, ALKBH5 stabilizes circEXOC5 demethylation while preventing YTHDF2 from binding to m6A-modified circEXOC5, thereby inhibiting its degradation. Upregulated circEXOC5 directly binds to insulin-like growth factor 2 mRNA binding protein 2 (IGF2BP2), promoting ATF3 mRNA degradation and ferroptosis, thereby exacerbating sepsis-induced ALI.^[^^56^^]^ A recent study demonstrated that ALKBH5-mediated m6A modification stabilizes the C—C motif chemokine ligand 1 mRNA. This stabilization promotes regulatory T cell recruitment, which exacerbates sepsis-induced ALI.^[^^57^^]^ This finding offers new insight into ALKBH5’s role in ALI and highlights a potential therapeutic target.

### m6A readers in ALI

#### YTHDF1

YTHDF1 modulated F-box protein 3 stability in an m6A-dependent manner, thereby affecting mitochondrial function and mitigating sepsis-induced ALI.^[^^58^^]^ In a recent study using LPS-treated RAW264.7 cells, YTHDF1 promotes guanylate-binding protein (GBP)4 expression by regulating m6A modification of GBP4 mRNA. This regulation affects M1 macrophage polarization and pro-inflammatory activity, promoting the release of cytokines such as TNF-α and IL-6, which damage alveolar capillaries and exacerbate the inflammatory response in ALI.^[^^59^^]^

#### IGF2BP3

IGF2BP3 is associated with ARDS owing to its role in tissue proliferation and apoptosis. Elevated IGFBP3 expression was also detected in fibrotic lung tissue and bronchoalveolar lavage fluid (BALF) from patients with ARDS and individuals with risk factors for ARDS.^[^^60^^]^ However, one study found that non-survivors of ARDS had significantly lower IGFBP3 levels compared to survivors. This discrepancy may have resulted from an inverse association between circulating and pulmonary IGFBP3 levels.^[^^61^^]^

## Therapeutic Prospects for m6A Modification

Currently, no preclinical studies or drug development efforts have been reported on the application of m6A modification in ALI treatment, and research remains in the exploratory stage. Recent experimental studies have shown that cellular damage in ALI can be effectively reduced by small interfering RNAs targeting key regulators of m6A methylation, such as METTL3 and METTL14.^[^^40^^,^^52^^]^ These preliminary findings offer valuable insights for future diagnostic and therapeutic research. These results suggested that strategies precisely regulating m6A methylation levels or targeting associated proteins could represent promising approaches for ALI diagnosis and treatment. Further rigorous and systematic research is required to validate the feasibility and efficacy of these strategies.

Currently, no approved drugs target m6A modifications; however, preclinical studies have identified several promising candidates. Research on m6A methylation in acute myeloid leukemia (AML) has advanced rapidly. Two selective METTL3 inhibitors, STM2457 and STC-15, exhibited potent anti-leukemic activity in preclinical AML models. Notably, STC-15, the first RNA methyltransferase inhibitor to enter clinical trials, is currently in a phase I trial (NCT05584111), opening a new avenue for targeted therapy.^[^[62], [63], [64]^]^ Other strategies include the FTO inhibitor FB23-2 and the IGF2BP2-targeting compound CWI1-2, both of which show anti-AML activity.^[^^65^^,^^66^^]^ Recently, the novel FTO-targeted meclofenamic acid-loaded nucleic acid nanodrug (SNAMA) inhibited intraocular melanoma by activating m6A-mediated disulfide death pathways, and its optimized form, SNAMA-apt, may enter clinical testing.^[^^67^^]^ In the respiratory system, m6A modifications have been linked to lung cancer progression. STM2457, an m6A modulator, was shown to regulate chemoresistance in small cell lung cancer (SCLC).^[^^68^^,^^69^^]^

Although targeted therapies for m6A methylation hold great potential, they face several major challenges. First, m6A regulates mRNA splicing, translation, and stability. Systemic targeting may cause off-target effects; for example, METTL3 inhibitors suppress tumor growth and damage normal hematopoietic stem cells, leading to myelosuppression. Second, m6A methylation is a dynamic and complex process involving various enzymes and cytokines. The same target may exert opposing effects at different pathological stages, complicating the timing of treatment. Finally, tumors can bypass m6A-targeted therapies through alternative pathways (e.g., other epigenetic changes), thereby worsening drug resistance.^[^[70], [71], [72]^]^ Targeting m6A methylation holds therapeutic potential. Despite preclinical advances, challenges such as target specificity, drug delivery efficiency, and resistance mechanisms remain unresolved. In the future, integrating multiomics analysis with Nano delivery technologies will be crucial for advancing precision therapies.

## Conclusions

m6A methylation is the most abundant internal epigenetic modification of RNA. Regulating m6A methylation holds significant therapeutic potential and may advance both general and pulmonary medicine. This review examined the role of m6A in ALI. Key m6A regulators, including METTL3, METTL14, and FTO, play central roles in AECs and macrophages. These modifiers control cell polarization, inflammation, ferroptosis, and autophagy during ALI progression. Elevated levels of m6A regulators have been detected in the BALF and plasma of patients with ALI/ARDS, consistent with findings in animal tissues. However, conflicting data remain; for example, METTL3 appears to exert both pro- and anti-inflammatory roles. Further research is needed to clarify context-dependent regulatory networks, particularly in human-derived samples. Future studies should incorporate larger sample sizes and more clinical specimens to address these challenges and support clinical translation. Comprehensive studies and clinical trials on m6A methylation are needed to advance the treatment of ALI.

## CRediT authorship contribution statement

**Yating Hu:** Writing – original draft. **Yijie Wang:** Resources. **Xiyue Liu:** Writing – review & editing. **Xiaoli Xue:** Writing – review & editing. **Binbin Li:** Writing – review & editing. **Fangwei Li:** Supervision.
